# Calciphylaxis in a Patient on Hemodialysis: A Case Report

**DOI:** 10.7759/cureus.74558

**Published:** 2024-11-27

**Authors:** Yahia Metwally, Hashim Abbas, Vishnu Jeyalan, Amjad Khanfar

**Affiliations:** 1 Nephrology, Manchester University NHS Foundation Trust, Manchester, GBR; 2 Cardiology, Manchester University NHS Foundation Trust, Manchester, GBR

**Keywords:** calciphylaxis, ckd, end stage renal disease (esrd), hemodialysis, penile calciphylaxis, serious complication of hemodialysis, sodium thiosulfate, uremic calciphylaxis, warfarin-induced calciphyalxis

## Abstract

Calciphylaxis is a rare but life-threatening complication of end-stage renal disease (ESRD), most often seen in patients undergoing hemodialysis. This condition is driven by calcium deposition in small blood vessels, leading to restricted blood flow, tissue ischemia, and often severe pain. While calciphylaxis typically affects areas with increased adiposity, such as the abdomen and proximal extremities, it can manifest on any skin surface, including rare sites like the genital region. Managing calciphylaxis is particularly challenging due to its rapid progression and limited treatment options. We report the case of a 53-year-old man with ESRD caused by long-standing diabetes, who developed painful necrotic lesions on the glans penis. Despite early diagnosis and treatment with sodium thiosulfate, his condition rapidly worsened. This case highlights the devastating progression of penile calciphylaxis and the complexities of managing such a rare presentation.

## Introduction

Chronic kidney disease (CKD) affects approximately 3.5 million individuals in the United Kingdom, with uncontrolled diabetes and hypertension being the primary etiologies. Among these, over 68,000 patients have progressed to kidney failure (stage 5 CKD), necessitating renal replacement therapies such as dialysis or transplantation. CKD significantly contributes to mortality, accounting for an estimated 40,000 to 45,000 deaths annually in the UK. Notably, individuals from Black, Asian, and minority ethnic groups are at a fivefold increased risk of developing CKD compared to other populations [1,2].

Calciphylaxis, also known as calcific uremic arteriolopathy, is a rare but devastating complication of CKD, particularly in patients with ESRD on hemodialysis. This condition involves calcification of small and medium-sized arterioles, leading to ischemic necrosis of the skin and subcutaneous tissues. While calciphylaxis commonly affects areas with abundant adipose tissue, such as the thighs and abdomen, penile involvement is exceedingly rare, accounting for approximately 6% of cases. Penile calciphylaxis carries a particularly poor prognosis, with mortality rates estimated at 50% within three months and 62.5% at six months [1].

The pathogenesis of calciphylaxis is multifactorial, involving disturbances in calcium-phosphate metabolism, hyperparathyroidism, and vascular calcification. Risk factors include diabetes mellitus, obesity, hyperphosphatemia, hypoalbuminemia, and the use of medications such as warfarin. Clinical presentation typically includes painful, violaceous skin lesions that may progress to necrotic ulcers [2,3]. Diagnosis is primarily clinical and management is usually challenging and often requires a multidisciplinary approach.

In this report, we present a case of penile calciphylaxis in a patient with ESRD secondary to diabetes mellitus, highlighting the clinical course, diagnostic challenges, and therapeutic interventions undertaken. This case underscores the aggressive nature of penile calciphylaxis and the need for heightened awareness and prompt management of this rare condition.

## Case presentation

A 69-year-old man with end-stage renal disease (ESRD) and atrial fibrillation presented to the dialysis unit with severe penile pain and lesions. Notably, he had visited the Emergency Department weeks earlier with similar symptoms and was evaluated by the urology team, but calciphylaxis was not initially considered as a potential diagnosis.

**Figure 1:**
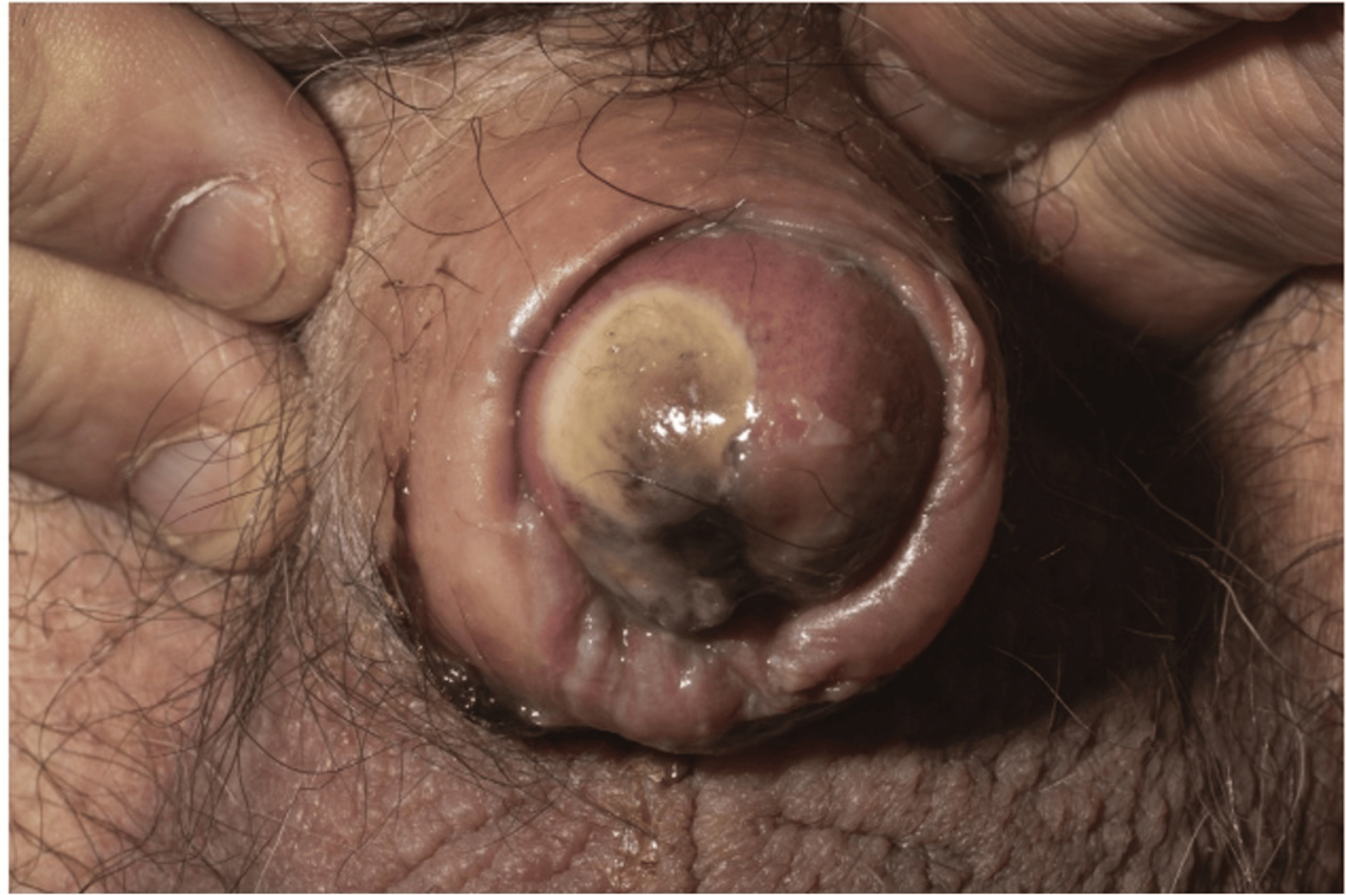
Painful penile lesions

**Figure 2:**
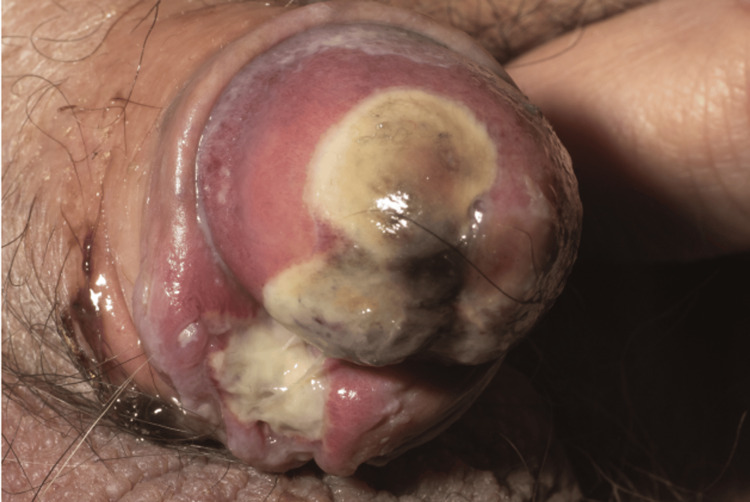
Painful and very tender penile lesions (Calciphylaxis).

On examination, painful necrotic lesions were observed on the penis, raising significant concern. A follow-up assessment a week later revealed rapid and alarming progression of the lesions (Figures 1-2), prompting the clinical team to strongly consider calciphylaxis. Treatment was initiated immediately.

The patient was taking warfarin for atrial fibrillation, a medication known to be associated with an increased risk of calciphylaxis. Given this, warfarin was promptly discontinued. This case highlights the importance of developing a risk assessment protocol for ESRD patients before starting warfarin therapy, as its use can inadvertently exacerbate already fragile vascular conditions.

To address the calciphylaxis, the patient was started on sodium thiosulfate during his dialysis sessions. Sodium thiosulfate is thought to work through a dual mechanism: chelating calcium ions to form a more soluble compound, calcium thiosulfate, which is easier for the body to clear, and acting as an antioxidant to reduce oxidative damage. This therapy is often considered first-line for calciphylaxis. Alternative treatments such as hyperbaric oxygen therapy, with or without surgical debridement of necrotic tissue, have also been reported in the literature, along with emerging options like apheresis and tissue plasminogen activators. However, these were not pursued in this case.

Despite receiving several sessions of sodium thiosulfate alongside dialysis, the patient’s condition continued to decline. Sadly, he passed away three weeks after his diagnosis. This tragic outcome highlights the significant cardiovascular burden and advanced vessel disease that often accompany this rare and devastating condition.

## Discussion

Penile calciphylaxis is a rare and severe manifestation of calcific uremic arteriolopathy, primarily affecting patients with ESRD undergoing hemodialysis [4,5]. It is characterized by calcification of small and medium-sized arterioles, resulting in ischemia and necrosis of penile tissue [6-8]. The prognosis for calciphylaxis is extremely poor, with mortality rates exceeding 50% within three months of diagnosis [1,8,9].

**Figure 3:**
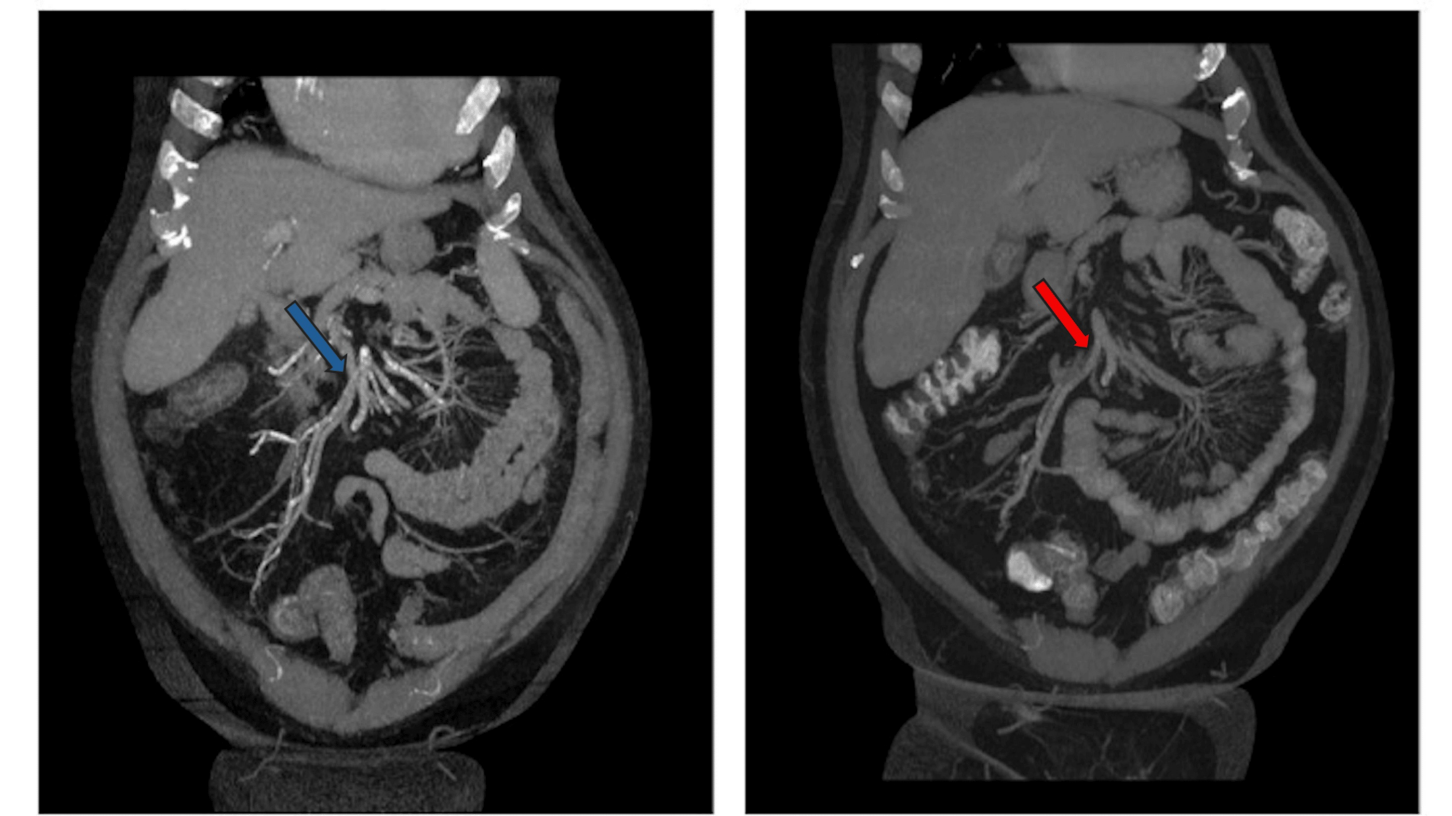
Computed Tomography (CT) abdomen and pelvis following intravenous contrast administration in portal venous phase. Images were obtained with a time difference of 3 years. On baseline examination (Right image), there was moderate diffuse calcified atherosclerosis affecting the abdominal aorta and main abdominal and pelvic branches (red arrow). Follow-up examination (left image) showed significant interval worsening especially involving splanchnic and mesenteric vasculature (blue arrow), bilateral iliac axis and deep pelvic branches.

**Figure 4:**
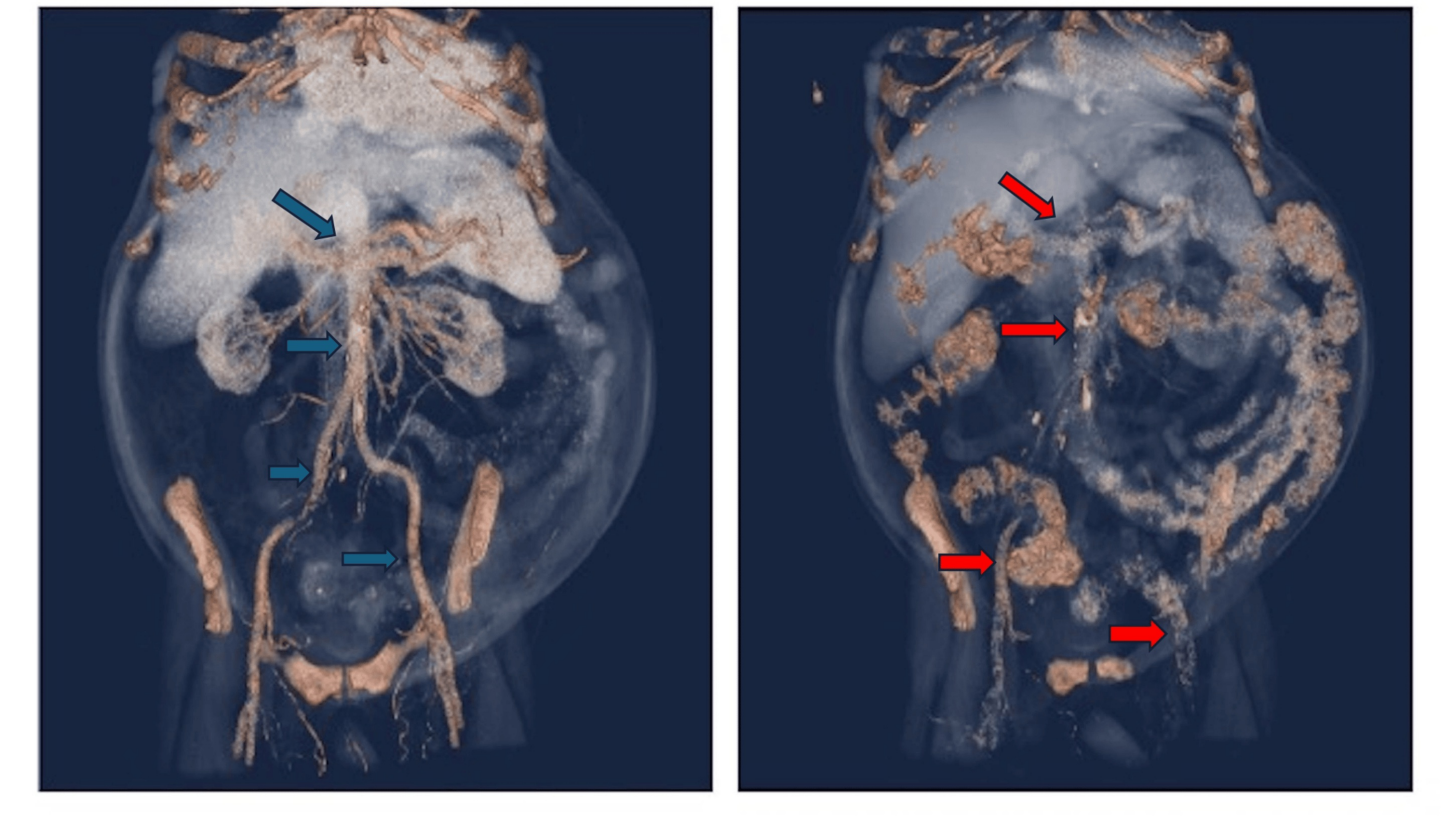
Coronal maximal intensity projection reformatted computed tomography (CT) and anterior projection 3-dimensional volume rendering. Images were obtained with a time difference of 3 years. On baseline examination (right image), there was moderate diffuse calcified atherosclerosis affecting the abdominal aorta and main abdominal and pelvic branches (red arrows). Follow-up examination (left image) showed significant interval worsening especially involving splanchnic and mesenteric vasculature, bilateral iliac axis and deep pelvic branches (blue arrows).

**Figure 5:**
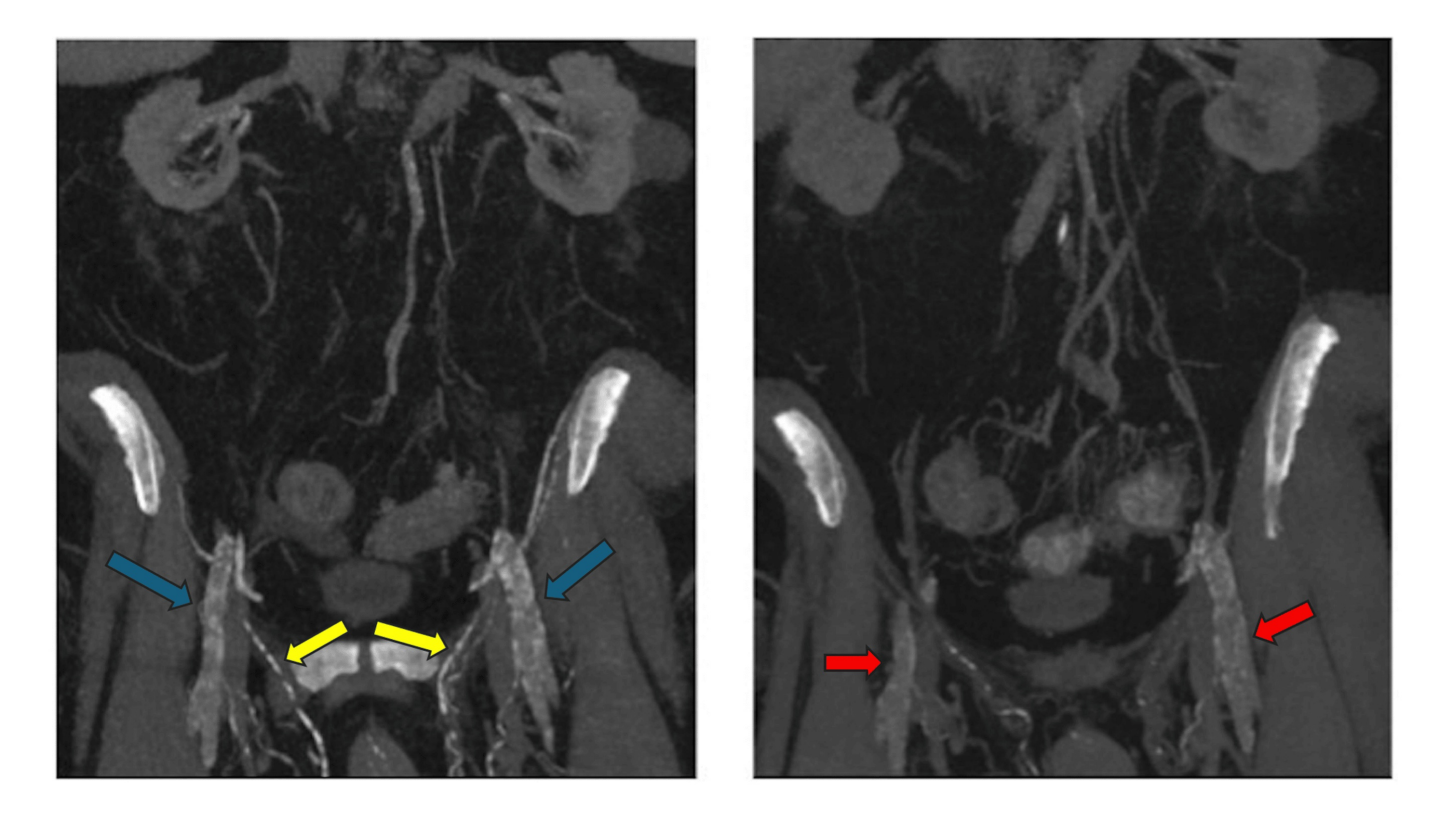
The coronal maximum intensity projection reformatted Computed Tomography (CT) images of the pelvis demonstrate significant interval progression of vascular calcifications over a three-year period. The image on the right represents the baseline examination, showing calcifications involving the bilateral external iliac, femoral (red arrows) and external pudendal arteries. In contrast, the image on the left illustrates marked worsening of calcifications in the bilateral external iliac, femoral (blue arrows), and external pudendal (yellow arrows) arteries; indicative of progressive vascular burden associated with the patient's condition.

**Figure 6:**
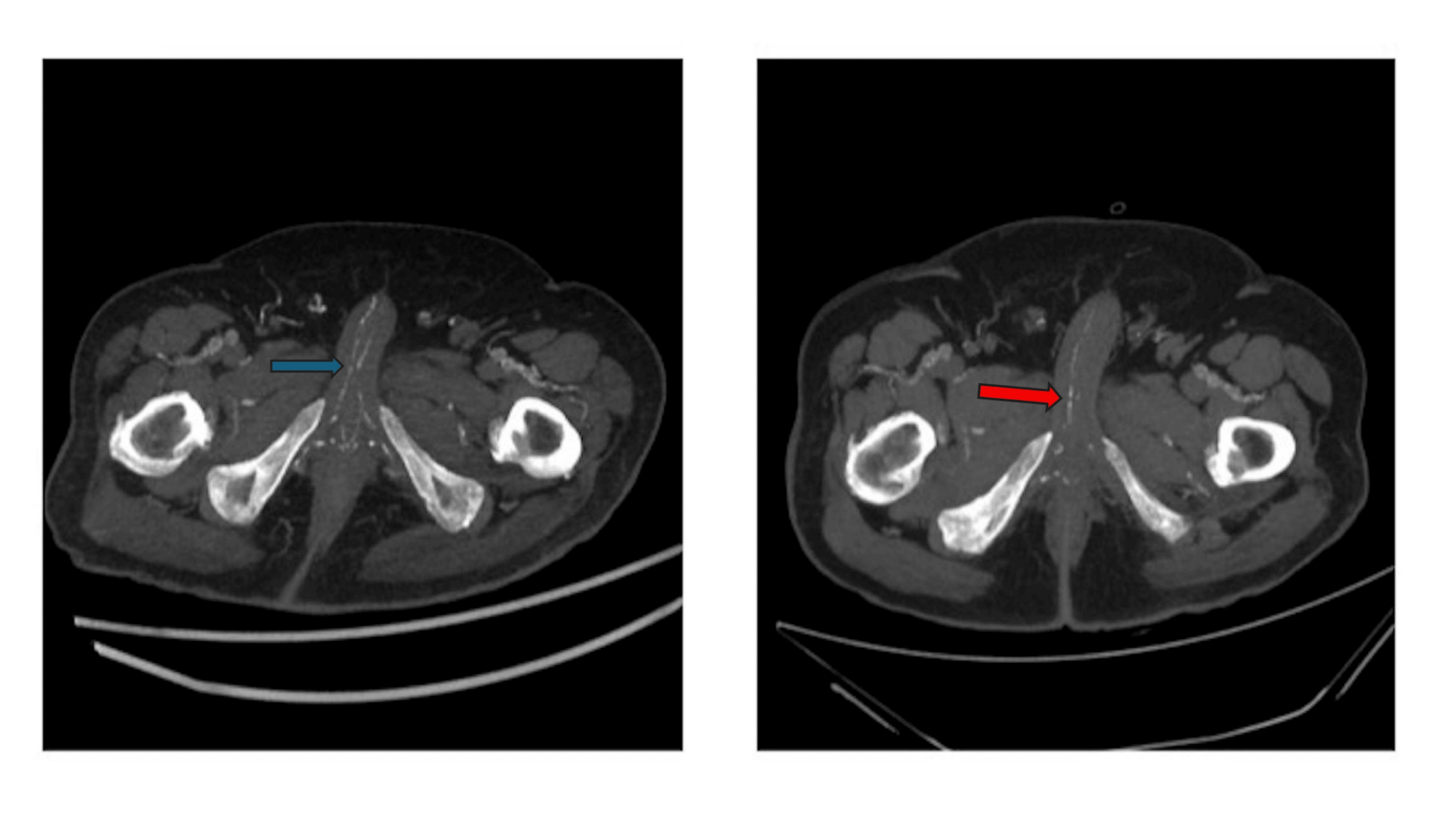
The axial maximum intensity projection reformatted computed tomography (CT) images reveal a significant interval worsening of calcifications in the penile arteries. The image on the left shows more pronounced calcifications in both the cavernosal and dorsal arteries of the penis (blue arrows), indicating disease progression when compared to the baseline examination on the right for the same arteries (red arrows).

The pathogenesis of calciphylaxis is multifactorial, involving disturbances in calcium-phosphate metabolism, secondary hyperparathyroidism, and extensive vascular calcification. Known risk factors include diabetes mellitus, obesity, hyperphosphatemia, hypoalbuminemia, and the use of medications such as warfarin [2,6,7,10,11]. In the presented case, the patient’s ESRD and warfarin therapy for atrial fibrillation likely contributed to the development of penile calciphylaxis. Images obtained for this patient over a three-year period demonstrated significant progression of vascular calcifications (Figures 3-6), underscoring the profound vascular burden associated with this disease.

Patients with penile calciphylaxis typically present with painful, violaceous skin lesions that may progress to necrotic ulcers [2,7,9,11]. Diagnosis is primarily clinical but can be supported by imaging studies that reveal vascular calcifications and, in some cases, skin biopsy to confirm the diagnosis [7,8,10,11]. In this case, the initial presentation of penile pain and lesions, followed by rapid necrotic progression, aligns with the typical clinical features of penile calciphylaxis.

Management of calciphylaxis is highly challenging and requires a multidisciplinary approach. Treatment strategies focus on wound care, pain management, discontinuation of offending agents such as warfarin [6,7,9], correction of metabolic abnormalities, and the use of therapeutic agents like sodium thiosulfate [6,7,10,12]. In specific cases, surgical debridement of necrotic tissue may be necessary [2,3]. For this patient, discontinuation of warfarin and initiation of sodium thiosulfate therapy were appropriate steps. However, despite these interventions, the condition progressed rapidly, reflecting the aggressive nature of penile calciphylaxis and the difficulties associated with its management.

The prognosis for penile calciphylaxis remains grim, with mortality rates estimated at 50% within three months and 62.5% at six months [1,8,9,12]. This patient’s rapid decline and death only three weeks after diagnosis highlight the devastating course of this rare disease. These findings stress the importance of early recognition, aggressive management, and the need for further research into more effective treatment strategies for calciphylaxis.

## Conclusions

This case addresses several important lessons in the management of calciphylaxis. First, it highlights the need for a robust risk assessment tool before initiating warfarin therapy in patients with ESRD. Given the well-documented association between warfarin and calciphylaxis, clinicians must carefully balance the benefits of anticoagulation with the risk of this life-threatening complication. Second, the importance of early recognition cannot be overstated. Although calciphylaxis is a rare condition, its consequences when unrecognized can be devastating. Clinicians must maintain a high index of suspicion for calciphylaxis when managing patients with kidney disease, including those with acute kidney injury, who present with new and painful skin lesions. A multidisciplinary diagnostic approach-including dermatologic evaluation, histopathological analysis, and radiographic tools-may be necessary to achieve a timely and accurate diagnosis. Finally, this case highlights the profound vascular burden associated with calciphylaxis, which reflects the severity of systemic disease when symptoms appear. Addressing this burden requires a multidisciplinary approach to management, including metabolic control, pain relief, wound care, and adjunctive therapies such as sodium thiosulfate. The integration of these strategies is crucial for optimizing outcomes in this challenging and often fatal condition.
